# Qualitative assessment of patients’ perspectives and needs from community pharmacists in substance use disorder management

**DOI:** 10.1186/s13011-021-00374-x

**Published:** 2021-05-01

**Authors:** Sarah Fatani, Daniel Bakke, Marcel D’Eon, Anas El-Aneed

**Affiliations:** 1 College of Pharmacy and Nutrition, University of Saskatchewan, 107 Wiggins Road, Room 3D01.3, Saskatoon, Saskatchewan S7N 5E5 Canada; 2 Education Innovation Institute, Medical College of Georgia, Augusta University, Augusta, USA

**Keywords:** Community pharmacists, Substance use disorder, Methadone, Qualitative study

## Abstract

**Background:**

Non-medical use of psychoactive substances is a common harmful behavior that leads to the development of Substance Use Disorders (SUDs). SUD is a significant health concern that causes adverse health consequences and elevates the economic burden on the health care system. SUD treatment plans that utilize a patient-centered approach have demonstrated improved treatment outcomes. It is essential for health care providers, including community pharmacists, to understand patients’ needs and prioritize them. Therefore, this study was conducted to explore the perspective of patients living with SUDs or who used substances non-medically regarding community pharmacist services and the delivery of services in a community pharmacy setting. The study took place in Saskatoon, a small urban center of Saskatchewan, Canada.

**Methods:**

Qualitative methodology was used for this research inquiry. Four focus groups were conducted, with a total of 20 individuals who had experienced substance use and accessed community pharmacy services. The discussion of the four focus groups was transcribed verbatim and analyzed independently by two researchers. Agreement on the emergent themes was reached through discussion between the two researchers.

**Results:**

Data analysis resulted in four themes that described participants’ perspectives about community pharmacists. The four emergent themes are: 1) conflicted experiences with community pharmacists, 2) lack of knowledge concerning community pharmacists’ extended services, 3) negative experiences in Opioid Agonist Therapy (OAT) program, and 4) needs from community pharmacists.

**Conclusion:**

There is significant potential for the patient-pharmacist relationship to address the varying needs of patients who use substances and improve their overall health care experience. Patients who use substances are receptive to pharmacists’ services beyond dispensary; however, respectful communication, provision of drug-related information, and counseling are among the primary demands. Future research should focus on studying the impact of meeting the needs of patients on their treatment outcomes.

## Introduction

Substance Use Disorders (SUDs) are a prevalent disease that affects an individual’s social, physical, and psychological wellbeing. Other difficulties usually co-occur with SUDs, such as legal and financial problems, loss of productivity, family instability, and unemployment [1]. Lost workplace productivity is a common consequence of substance use and SUDs. In 2014, the estimated loss of productivity in Canada due to substance use was estimated at $15.7 billion, with tobacco, alcohol, and opioids responsible for most losses [2]. These social situations are both symptoms of SUDs and causes that exacerbate the disease. Other SUD-related problems vary among patients in terms of symptoms, intensity, and responsiveness to treatments [3]. For example, Relapses, a common aspect of SUDs recovery, can have varying triggers depending on the individual’s experience with substance use [4, 5]. Therefore, it is integral that tailored treatments are provided for patients living with SUDs that address their etiological and symptomatic variations in a patient-centered approach. Addressing the needs of patients living with SUD, beyond the pharmacological aspects, has been proven to reduce substance use [6, 7]. Health care providers, such as physicians and pharmacists, who interact with SUDs patients, need to acknowledge the positive impact of patient-centered care and prioritize patients’ needs in treatment decisions.

Community pharmacists are among the health care providers who most frequently encounter clients living with SUDs. In Canada, community pharmacists are the main providers of methadone, the primary treatment of Opioid Use Disorder (OUD). Community pharmacies provide Opioid Agonist Treatment (OAT) programs that primarily dispense oral methadone to facilitate patients’ accessibility to the therapy. Pharmacists working in a community pharmacy with OAT programs are uniquely positioned to encounter patients with SUDs and who use substances for non-medical reasons on a daily basis. In Canada, community pharmacists’ roles in OAT include dispensing and witnessing patients’ consumption of a prescribed dose of methadone.

Easy access and extended working hours make community pharmacists among the most accessible health care providers in Canada. Proximity and accessibility is an impactful combination to initiate and sustain effective strategies to address SUDs [8]. Pharmacists’ accessibility must also be utilized to deliver substance use preventative services like screening and referral. Community pharmacists have been successful in achieving the goals of many health initiatives, such as smoking cessation [9] and diabetes management [10]. Therefore, we would expect that involving community pharmacists in substance use preventative services would help manage and alleviate the negative consequences associated with substance use [11–13]. Utilizing community pharmacists in harm reduction and preventative services for patients with SUDs is not novel; however, not yet fully exploited [14].

Therefore, it is critical to closely investigate the relationships between community pharmacists and pharmacy clients who use substances, to recognize the facilitators and barriers towards providing preventative initiatives in community pharmacies. A study by Vorobjov et al., investigated pharmacists’ perspectives regarding substance use and providing services for people who use substances. The study found that pharmacists are willing to be educators for the public and provide preventative services for clients who use substances [15]. Some pharmacists also recognized opportunities in daily practice to intervene and help clients in the early stages of SUDs [16]. On the other hand, pharmacy customers with risky alcohol consumption had a positive attitude regarding utilizing alcohol screening services provided by their regular community pharmacists [17]. In contrary, people who inject drugs reported stigmatized encounters with community pharmacists when purchasing clean needles from community pharmacies [18, 19]. Thus, the perspectives of patients who use substances regarding community pharmacists’ services must be further examined so that the provided services match patients’ needs [20–22], while applying patient-centered care for patients living with SUDs.

In a continuation to our past work in which we probed pharmacists needs [16], in this study the perspectives of patients who use substances non-medically, or living with SUDs, are explored regarding community pharmacist services and the delivery of care in a community pharmacy setting. The purpose of the study was to 1) explore patients’ perceptions regarding community pharmacists’ delivery of services, including harm reduction, counseling, and referral to community/social services; 2) identify available services and resources for patients who use substances in community pharmacies; 3) identify types of services and resources that are not provided by community pharmacists which patients want to receive, and 4) explore the barriers patients face while accessing care in a community pharmacy.

## Methods

### Setting, recruitment, and selection criteria

The study was conducted in the city of Saskatoon, Saskatchewan, Canada. Saskatoon is the largest city in the province of Saskatchewan (population of approximately 275,000) and is known for its high rate of HIV due to substance use [23, 24]. Purposive and snowball recruitment for people who use substances was conducted with support from community-based and harm reduction organizations that have regular contact with people who use substances, namely AIDS Saskatoon, currently named Prairie Harm Reduction (https://prairiehr.ca/) and Station 20. Purposive recruitment is popular in qualitative studies to yield informative cases for the phenomena under study. However, it also limits control over the sample criteria such as age and education [25]. A poster outlining the research was distributed throughout the organizations from June – July 2016. Participants had to be individuals over 18 and current substance users, or individuals who had engaged in non-medical substance use in the past 2 years (illicit drugs and/or prescription drugs), and accessed community pharmacy services. Ethical approval was obtained from the University of Saskatchewan Ethics Board (Beh#16–256).

### Data collection

A qualitative methodology was considered the appropriate approach for this research project. Qualitative studies are used to explore and understand in-depth human-related phenomena [26]. Qualitative research is used to understand the participants’ lived experiences and develop patterns and relationships between different constructs. The focus group was the chosen method for data collection due to its effectiveness in exploratory studies, especially when interaction among individuals is needed to facilitate discussion and yield a broader range of ideas [27–29]. Focus groups have become one of the most frequently utilized data collection methods in primary research [30, 31]. A discussion guide was developed and reviewed by two researchers of the research team to ensure validity and relevance to the research objectives. The discussion guide was developed to facilitate the conversation in the focus groups. The questions were carefully designed to lead the discussion towards the areas of interest (Table 1). There was a mixture of open and closed-ended questions that were tailored to be sensitive, clear, and unbiased.

**Table 1:** Focus Group Discussion Guide

Clarification of Terms	1) People Who Use Drugs 2) Community Pharmacist 3) Pharmacy 4) Any Others?
Introductory Question	1) What experiences have you had speaking with community pharmacists?
Transition Questions	1) Can you identify services and information that are available from a Pharmacist for people who use drugs? 2) Is a pharmacist a good person to get health information or services from?
Key Questions	1) Think back to a time that you’ve visited a pharmacy in the past and spoke with a pharmacist. Were there any specific reasons why you did or did not feel comfortable speaking to a pharmacist about your care? 2) When you visited the pharmacist, what were the most helpful services or information that a pharmacist provided you? 3) Pharmacists in some countries are able to offer needle exchange, information about HIV or HCV or dispense naloxone. What would be the most helpful services or information that a pharmacist should provide that they don’t have now?
Final Question	Is there anything else that anyone feels that we should talk about but didn’t?

### Focus group procedure

For participants’ convenience, the focus groups took place at a private conference room in a community enterprise center (Station 20) in the west-side core neighborhoods of Saskatoon. All participants signed consent forms and completed a brief survey to verify their eligibility before the focus group. The participants had the chance to ask questions after the consent form was read and explained to them. Participants were informed about the research’s confidentiality, and that no data could be identified or linked to a specific participant. Also, participants were asked to maintain the privacy of other participants and not share members’ information with non-participants.

The focus group discussions were structured using the developed guide to maintain consistency across the multiple focus groups. At least one researcher acted as the moderator to prompt and engage participants in productive discussions during the focus groups while using the questions guide to maintain focus. At the end of each focus group, participants received honoraria ($25 Tim Hortons [a coffee shop chain] gift cards) for their participation in the research.

### Data analysis

Four focus groups were conducted with a total of 20 participants (8 females and 12 males) for an average of 1 h per session. All the participants identified themselves as people who use/used substances nonmedically. No participants withdrew from the study. Participants were divided as follows: focus group 1 (7 participants); focus group 2 (4 participants); focus group 3 (5 participants); focus group 4 (4 participants). The age categories of participants were: one participant in the category of 18–24 years old; three participants in the category 25–34 years old; eight participants in the category of 35–44 years old; five participants in the category of 45–54 years old; and three participants in the category of 55–64 years old. Although it was not required, two participants identified themselves as HIV positive, and one participant identified with a first nation status. All participants but one were enrolled in the Opioid Agonist Therapy (OAT) program using oral methadone doses. OAT program in Canada is administered through community pharmacies to treat patients with Opioid Use Disorder (OUD) using several drugs, such as methadone and buprenorphine-naloxone. Community pharmacists must dispense and witness patients consume their daily doses of methadone. Having almost all participants enrolled in the OAT program unintentionally biased the sample recruited for the study.

The number of focus groups was predetermined to be four for this study, as a range of 3–5 participants per group is considered an acceptable range [32]. After four focus groups, saturation was reached, and no further groups were needed [32–34]. The focus groups were audio-recorded, then transcribed verbatim. Using NVivo software, inductive content analysis was performed independently by a research team member (SF) and a research assistant [35]. The latter was hired as an external researcher to reduce bias and ensure the validity of the analysis. The analysis started with repeated reading of the data transcripts to interpret the data as a whole. The initial coding system looked for all ideas and concepts that were described throughout the data. Similar and related codes were grouped into several sub-categories. The sub-categories were combined into higher-level categories (i.e., generic categories) [35]. Generic categories are the main themes represented in the results. Each researcher conducted these steps independently. After completing their independent analysis, the two researchers met to compare their coding systems and emergent themes. This iterative process was conducted until an agreement on the main categories was reached. Emerged themes were reported in all focus groups.

## Results

Four major themes emerged, and the analysis revealed several recommendations to improve community pharmacy services for patients living with SUDs. The four themes provided insights regarding participants’ experiences and perceptions of community pharmacists as health care providers. They also summarized the participants’ needs that went beyond the pharmacological aspects of treatment. The themes also explained the effect of physical space and the community pharmacy setting on the communication between pharmacists and those with SUDs. Finally, the developed themes described participants’ experiences with the services provided by community pharmacists, particularly harm reduction services, such as needle exchange and maintenance therapy of methadone.

### Conflicted experience with community pharmacists

Through daily and weekly visits to community pharmacies, all participants had notable experiences with community pharmacists.. Participants’ visits to community pharmacies were mainly to obtain their methadone and HIV medications. They described community pharmacists as the most seen health care provider, indicating that pharmacists are nearby and have long working hours making them accessible. Paradoxically, pharmacists were described by participants as the health care providers they would be least likely to seek out when help was needed. They elaborated by saying pharmacists have a busy work environment and hardly reply to their questions or consultations.

*“Participant A - ‘ I never really thought about going to him [referring to community pharmacist] for support for anything. I always just thought of him as someone serving me just to get the pills and … And, get out.’*

*Participant B – ‘Get your drugs, and that's it.’"*

The main aspects that shaped participants experiences with community pharmacists are listed as sub-themes:

#### Time

Lack of time was a core problem often reported by participants. They elaborated that community pharmacists are always busy and do not have time to communicate with their patients. Lack of time for appropriate communication, being too busy, and multitasking were common themes across all group meetings. Lack of time was the main reason that participants did not seek medical advice, or help from community pharmacists. Participants expressed that pharmacists did not have time in their daily routine to provide one-to-one counseling for them.

*" Probably we don't go to them, like for counseling or whatever because it seems like they don't have enough time, [it] seems like they're so busy.*

*Answering phones for the next prescription. They don't have time to even look at you. And, then when you do get a chance, they'll start and then … Oh, excuse me, I got to get going’."*

#### Profitability

Participants explained that some community pharmacists could be helpful when they have time, but the business model of community pharmacy pushes pharmacists toward profitability. Profitability is mandated by the companies and forces pharmacists to “push drugs” and limit counseling time with their patients, as explained by participants.

*"..They have very, very, very little time to spend with patients. As a matter of fact, zero time to spend with patients because their job is to push drugs."*

Also, a number of participants articulated that pharmacists are multitaskers as they do not have time to properly counsel patients and provide medication-related information. They perceived that the extent to which pharmacists are required to multitask was harmful to patients’ care, resulting in suboptimal services. For example, several participants reported that the concentration of methadone varies from one time to another because pharmacists are multitasking and cannot stay focused on a specific task.

*" Yeah, in [Pharmacy Name and Location], like that's a constant problem with me too. There's always a different pharmacist filling [the] bottles. There's never one person, and it's never the same. I'm always finding my methadone's too, too much.’"*

*" You know, instead of having four different pharmacists doing one methadone … you know methadone for all these people, yeah. It's never constant, and it's always, yeah, I, I find sometimes that my, my methadone's too weak and I get that bone rot.’"*

According to the participants, community pharmacists are *“overworked, underpaid*” workers who are pushed by their companies to make a profit. Having this perspective of community pharmacy services affected patients’ engagement with pharmacists as they believe pharmacy services is a money-making business that endeavour to maintain power and control over the patients they serve.

*"I truly think it's a power/control issue. Extremely, that's what it's become."*

#### Consistency

Participants identified that lack of consistency in pharmacy services negatively affected their experiences in community pharmacies. Participants reported that dealing with a different pharmacist each time prohibits building trust and meaningful relationships with pharmacists. Having different pharmacists providing regular services like methadone, HIV, and Hepatitis C medication prevented the feeling of familiarity with the system and alienated patients.

*"Yes, that's exactly what the problem [is] … There's always a different pharmacist doing something. Right, one pharmacist for methadone, period. That would be great. That way that pharmacist knows who's, what, where, how, when and why."*

Lack of consistency in pharmacy procedures was also a concern for participants. For example, it was not clear for participants why some pharmacies would provide them with over-the-counter medications that contains opioid, such as a medication that contains acetaminophen with codeine (an opioid), while other pharmacies would deny it. Similarly, it was upsetting for participants that providing pamphlets containing information about their medications and illnesses was not a regular practice at all pharmacies. The following conversation took place in one of the focus groups:

*"Participant A – ‘You know what is weird … because of my methadone, uh, some pharmacies will sell me ones* [a medication that contains codeine, an opioid]*, and some other pharmacies will not sell me ones.’*

*Participant B – ‘Yeah, I am wondering how come other pharmacies will sell me them? And, I have asked them too, like, why do you guys do this … like, are you purposely giving me a hard time? Because other pharmacies do it, no problem.’"*

Participants suggested pharmacies hire more pharmacists to improve services. Participants believed that if pharmacies were properly staffed, pharmacists would have more time to answer questions, improve consistency, and augment the quality of the provided services.

*"You know, it's, it's constantly like that. That's why if they got one pharmacist for the methadone, HIV, Hep C, whatever. Then, that pharmacist is just doing that job and able to answer questions for you. Then, that, I would feel much better about all pharmacists. But right now, when go I see my pharmacist, I got no time for them either. Cause, why? They don't, they look at you, they sneer their nose down at you, or whatever and then have a nice day."*

#### Positive encounters

An interesting observation occurred when participants from different focus groups shared positive stories of the same pharmacists. A number of pharmacists, known by name, were able to provide a positive experience to multiple participants. Polite, genuine, friendly, and caring were the main characteristics of the pharmacists who created positive experiences for participants. Participants perceived that those pharmacists sincerely cared, tried to put their patients' health first, and never let them go without the necessary medications. The following conversation took place in one of the focus groups:

*"Participant A –‘certain pharmacies are good, like, it depends on the pharmacist. Like, [Pharmacist Name] was an awesome pharmacist … ’*

*Interviewer – ‘What, what makes [Pharmacist Name], a good pharmacist?’*

*Participant A – ‘Well, his attitude.’*

*Participant B – ‘He cared about people.’*

*Participants A – ‘He actually cared.’*

*Participant C – ‘He, like, he mingled with the people, you know.’*

*Participant A – ‘He never ever let me go without my medication.’"*

### Lack of knowledge concerning community pharmacists’ extended services

Participants showed a lack of knowledge regarding the scope of services community pharmacists could provide. It was surprising for participants that community pharmacists are able to provide services beyond dispensing medications. The role community pharmacists provided for them has been limited to the dispensing of medications, namely methadone and HIV medications. However, even the dispensary services were reported as suboptimal because of pharmacists’ multitasking and poor communication.

*"I always just thought of him as someone serving me just to get the pills and get out."*

While another participant mentioned:

*"The only reason, the only reason I use a pharmacist … they go get my, my product."*

The limited understanding of community pharmacists’ roles, services, and responsibilities created a communication barrier for the patients when accessing pharmacy services. A lack of knowledge about the services pharmacists provide discouraged participants from seeking help and other services beyond dispensing medication. When a pharmacist offered additional services such as dose adjustment or medical advice regarding their drug regimen, participants felt annoyed as they believed pharmacists were overstepping their role and delaying the dispensary services. Participants stated that understanding the broad scope of a pharmacist’s role would facilitate information exchange between patients and pharmacists.

*"I did not even know that they could do all that stuff because … they never … showed that they could do all that stuff."*

Another participant reported:

*"And, the pharmacist will sometimes say that, uh, I really do not think you need this medication, or it should be lowered, or something like that. And I do not think that it is their job to be doing that and when it comes to the doctor … I mean, when the doctor prescribes it, they should just be following what the doctor orders."*

However, once participants became aware of the impact community pharmacists could provide, they showed interest in accessing additional services if offered by community pharmacists. Some participants indicated that their pharmacy uses posters to promote different services they provide. Stronger relationships appeared to be formed between patients and pharmacists when patients accessed additional services via community pharmacies.

*‘Well, like at my pharmacy, they post up posters........,*

*Like, even when I've been sick when it happened on a weekend when it hasn't been a doctor, they can look up stuff that … if they can give me something, they can give me something until I can get to a doctor. I think I've been through that system three times already and I think it's pretty good.’*

Another participants added

*‘Yeah, that's how [Pharmacy Name] in Winnipeg … when I was in Winnipeg, that's how they were. Like, they're very like one-on-one basis. Like, they care for you. like they knew when something was wrong with me and they come up to me. Because I have mental health issues. And, so they know, like, I'd be off balance and stuff like that.’"*

Compared with other pharmacies in the vicinity, one specific Saskatoon pharmacy was identified across all focus groups as offering multiple services, resulting in a higher number of positive comments. Most participants recognized the “one-stop-shop” as convenient as most of them do not have transportation. It was the only community pharmacy in Saskatoon that has a unique patient-friendly arrangement, whereby participants were able to receive more services than what a usual pharmacy provides, including access to a nurse practitioner, a doctor 2 days a week, and a counselor. The pharmacists working in this pharmacy were also able to provide a positive experience for most of the participants.

*"In my pharmacy my methadone doctor comes on Tuesday [s]. And, we got nurse practitioners, in the building. And, it's open all week too. Other things like talk to the counselors there, and … we have all of that [Pharmacy Name]."*

### Negative experiences in OAT program

Enrollment in an OAT program was the predominant reason why participants accessed community pharmacy services. Almost all participants were enrolled in an OAT program, and those experiences appeared to shape their perspective of community pharmacists. Participants were glad to have the advantage of accessing the program that helped them manage their SUDs. However, the way the OAT program was operated generated negative feelings and experiences among most participants. Several participants showed frustration with the idea of methadone as a lifelong commitment. They believed that it creates a power disparity where pharmacists *“control how [their] health is right now.”*

The negative experiences about methadone were centered around pharmacists’ attitudes, pharmacy settings, and unclear procedures.

#### Pharmacists’ attitude

Although participants reported a few positive encounters, most of the participants’ comments were characterized as unfavorable, describing both stigma and discrimination. Participants felt that pharmacists’ attitudes showed prejudice. Those negative feelings were combined with participants’ beliefs that pharmacists do not understand the hardship they are going through to stay on the OAT program. They explained the difficult lifestyle they have as drug users and the significant effects of unforeseen events like a death in the family or a house fire. Despite all their sufferings, patients felt that pharmacists did not offer proper assistance and are finding different reasons every time they visit to cut them off methadone. For example, losing methadone bottles, coming late to their methadone appointment, being rude to pharmacy staff have been reasons cited by a pharmacist to refuse to give them their methadone dose. Participants believed that community pharmacists lack sincere compassion and enact barriers for them because they are on methadone.

*"Right. And, you got these people looking at you. Oh, you're on the methadone program. You're a user. You're garbage. That's how you feel because that's the response you get from all the people."*

Another participant elaborated on how they felt abandoned

*"No, health region helping us. I have some people hitchhiking on the highway, coming to get their methadone because the pharmacist won't give them a week or two days or three days."*

A third participant explained how pharmacists can’t feel them

*“Because they haven’t been in my shoes before, they don’t know what I’m going through. They’re not … they read books and think that they know everything.”*

Participants also believed that they are being discriminated against and treated differently than other clients because they are engaged with the methadone. Participants reported that being on methadone or having HIV or Hepatitis C evokes negative attitudes and behaviors from community pharmacists. Participants expressed that pharmacists’ body language changes once they know that a patient is a methadone client. For example, they feel because they are methadone clients, they are ignored, stigmatized, and pushed aside in favor of serving other clients.

***"*** *We shouldn't feel discriminated against because we're sick … and, a lot of these pharmacists do that. They will look at you, … like if they know that you're Hep C or HIV. Right, they're automatically … just will not touch you. … their language, … eye appearance. … Their facial expression.”*

Participants recognize how challenging the work environment is in community pharmacies. They explained that pharmacists provide services to a wide range of clients and that some methadone clients are rude and obstinate. They understand how stressful it is for pharmacists to validate the information provided by a SUDs patient as some patients may provide misleading information to break the rules. It was recognized that pharmacists’ behavior might be justified based on previous negative encounters with other methadone clients. However, participants felt that pharmacists should not judge all methadone clients negatively and should “*treat people how [they] want to be treated.*”

#### Pharmacy settings

Pharmacy settings for methadone patients were described as unwelcoming environments that made them feel uncomfortable, especially with pharmacies that designated a separate entrance and space for methadone patients. Using a different back door and dealing with pharmacists through a glass barrier was an upsetting experience for participants. Participants felt alienated because they had to access their services differently than other clients. In other pharmacies, the situation is less traumatic, but methadone clients were still treated differently and “pushed” aside to wait, unlike other clients.

*" … even if they had some pictures up, behind [Pharmacy Name], in the back door … there's nothing to make that person feel comfortable. You are in the cold; you're in an enclosed space that's no bigger than this (Participant indicates the size of the space by tracing it out in the room). With a, with a big plexiglass window and that is all you got. There are no pictures. It is always gross on the floor, and you feel like you're in a prisoner's box"*

Participants indicated that despite their negative experiences regarding a particular pharmacy setting or pharmacist’s attitude, they did not have the option of getting methadone from another pharmacy. They were forced by their SUDs and their doctors’ referrals to access certain pharmacies. Due to their SUD condition, they needed to utilize a nearby pharmacy, especially when they did not feel well.

Furthermore, the lack of privacy was reported as a communication barrier by different participants. Participants did not like to discuss and share sensitive information about their substance use with community pharmacists in such a public setting. Also, a few participants felt ashamed when a pharmacist discussed their medications and health concerns where others could hear. This behavior was perceived as a breach of confidentiality.

#### Procedures and policies of OAT

Unpredictability and lack of consistency in the procedures for methadone dispensing was a primary concern for several participants. Various reasons were shared as to why some pharmacists may refuse to dispense methadone, such as being late for appointments, being rude to the pharmacist, missing daily methadone doses for a couple of days, and losing carries bottles. While other pharmacists will provide methadone under the same circumstances as being late for appointment. The worst scenario was when, according to some participants, pharmacists refused to dispense medications without any explanation. Also, participants reported that pharmacists did not assist participants when they have unforeseen events like travel arrangements to attend an unexpected family funeral. It was not clear for participants what the policies were under such circumstances as pharmacists often lacked consistency in such situations. Participants theorized that pharmacists were prejudiced and enforced policy without caring about their patients.

Participants shared incidents of when they suffered from withdrawal symptoms after receiving their witnessed daily dose of methadone due to inaccurate dosing or because they vomited the dose. Participants explained how they felt abandoned as pharmacists did not help while witnessing their suffering. They explained the pharmacists did not replace their dose until they contacted a doctor. The situation sometimes resulted in the replacement of the methadone dose; however, other times, participants’ doses were not replaced. Participants felt controlled by pharmacists who may find different reasons not to give them their methadone carries – take-home methadone doses - or even their daily witnessed dose. It was upsetting for participants that pharmacists lacked compassion when enforcing policies and regulations.

### Needs from community pharmacists

Participants’ responses aggregated around three main aspects concerning their needs, namely respect, education, and the needle exchange program.

#### Respect

Participants explained that they wanted to receive respectful communication from community pharmacists, similar to other clients. They expressed that they deserved to be treated with respect, politeness, and care and not judged because of their SUDs. Participants also described that they would appreciate it if pharmacists socialized and engaged in friendly exchanges with them. Genuine understanding and respectful communication were the paramount need reported by participants. However, participants also clarified that it might take some time to build trust and form a relationship with them; therefore, the best way to interact with SUDs patients is to be professional, polite, and “*do not force it*.”

*"If I [were] a pharmacist, I would look at each individual case separately and would not judge a person if they are having a bad day. I would ask them, are you ok? I would direct them, you know, if you need someone to talk to, here is a number, you can go here. There is a job there, you know. There is a lot of help out there; you just got to reach out."*

Finally, participants wanted pharmacists to be sensitive to different cultures, languages, and practices, particularly the culture of the Indigenous peoples of Canada. An Indigenous participant described how great the experience would be for an Indigenous patient if a pharmacist showed a sign of cultural admiration.

*"They (referring to First Nations peoples) are all flown here to get their drugs. And, they are just, it is intimidating. It is scary as hell, having a pharmacist there, the pharmaceutical company ready to hop on you. They got a whole team of doctors as soon as they get off the plane. Not one of them speaks their language."*

#### Education

Participants acknowledged the need to be educated by community pharmacists regarding their medications, such as toxicity, drug-drug interaction, and drug-food interaction. Health information provision and explanations were one of the main topics discussed across all four focus groups. Learning about SUDs and understanding the effect of the medications on functionality was reported as an essential need. A pamphlet (print-out) with information about SUDs or where to get help was recognized as a great approach to providing information.

*"But they got to educate people more … and give them more information about the drugs people are taking. Instead of just prescribing and giving the person their prescription and, you know, go home and take your meds until they are done. And if they are prescribed Dilaudid or morphine, well, two weeks down the road they have no energy, they are sore, and they are sweating and everything. … they do not understand why because they did not get the information from the pharmacy when they started taking this."*

Another participant elaborated

*"what drugs interact with each other. I think it is a good idea that they should bring it to the drug addict's attention. Like say, for instance, this seizure medication combined with this medication, if you are abusing crystal-meth, it will do this to you, just a heads-up, … people just think that they are drug addicts and they do not care, who cares to let them know, but it's important because some of us are diabetic or suffering with mental illnesses like depression"*

Similarly, participants wanted community pharmacists to learn about the difficulties and social hardships they are going through as substance users. Many participants expressed that they wanted pharmacists to understand how hard it was for them to secure basic needs like food, shelter, and transportation. Participants also believed that community pharmacists needed more education and training on SUDs. According to the participants, pharmacists had a knowledge gap concerning SUDs and HIV; thus, they needed to be further educated in order to better serve patients with SUDs.

*"They do not know what we are talking about when I am discussing my lab results with them. CD4 count and … viral load. They did not know any of that. You know, just looked at me. They are real puzzled."*

#### The needle exchange program

Several participants described the city’s needle exchange program as a non-effective program because of how it was operated. They elaborated that the operating hours, the quantity of provided syringes, in addition to the limitations of the exchange policy, made the program ineffective when needed. Community pharmacies were not currently providers of the needle exchange program in Saskatoon; however, participants believed they should be. Having community pharmacists involved in the distribution of clean needles would enhance the accessibility of the program, especially on weekends.

"The Health Bus* is done at 11. After 11 and on weekends, you are done. If you do not have a clean rig, well, all of a sudden, you are using one of your used ones. Heaven forbid you would use somebody else's, but I am sure you would not in this day and age. Or, you are sharpening one of yours. I am it is, it is really quite gross. I could go into it. or [Pharmacy Name] could have it … … . could have a mandate of giving out five."

* Health Bus is a mobile health initiative in Saskatoon. It is designed to bring health care services to people and is staffed with nurse practitioners and paramedics.

## Discussion

This study’s main goal was to understand the perspectives and experiences of patients living with SUDs about community pharmacists as health care providers. The study’s findings suggested that the participants’ experiences with community pharmacists are generally negative, with many interactions lacking proper counseling and support. Similar to other studies, lack of pharmacist time [36], stigma [37], lack of privacy [38], and need for additional education for pharmacists [16] were mentioned by participants among the barriers to accessing care in a community pharmacy setting. Some of these issues may overlap with the needs of other customers out of the SUD population. However, understanding SUD patients’ needs are required so that changes within the pharmacy profession is driven by evidence to eventually change the culture impacting SUD patients. We acknowledge that the business model is playing a key role in the patient-pharmacist interactions; however, this is out of the scope of the study and will to be investigated in a future study.

Supporting the notion that the public is often unaware of pharmacists’ full spectrum of services [39, 40], participants shared their limited understanding of community pharmacists’ role beyond dispensing. Community pharmacists are not practicing the full potential of their role in caring for people with SUDs; therefore, it is not recognized by pharmacy consumers [41]. Community pharmacists are trained to provide extended services beyond the traditional model of pharmacy that focuses on filling and dispensing medications [42]. However, many barriers are hindering pharmacists from practicing their full capacity. Participants reported pharmacists’ lack of time as the main reason for not recognizing or accessing pharmacists’ extended services. Lack of time was also reported in other studies [36]. Pharmacist-patients relationship is “ritualised.” “Ritualized” relationship means that it involves well-known typical interactions. Based on previous encounters, pharmacists’ consumers, including people living with SUDs or consume substances, may not expect advice, help, or counseling from a pharmacist concerning their conditions, making it hard for pharmacists to initiate a conversation and change the ritual. Likewise, it may be challenging for patients to understand pharmacists’ extended role and seek their assistance [43]. To change the encounters between patients and pharmacists, the business model, guidelines and policies should encourage pharmacists to practice their responsibilities and set proper remuneration for such services [44]. Pharmacists’ roles and responsibilities should be clearly defined for pharmacists and promoted among patients. Also, to change or enhance this relationship, environmental contextual cues like promotional posters and pamphlets would encourage pharmacists and patients to utilize these services, as reported previously [45].

While patients’ satisfaction is an integral element for patients’ retention and adherence to the OAT program [46], the overall experience of participants with the OAT program in Saskatoon was mostly unfavorable. Poor communication, an unwelcoming environment, lack of counseling, and lack of proper information were the primary reasons cited by participants for their negative experiences. Also, participants reported that the lack of transparency in the OAT program policy and procedure added another layer of communication barrier. In fact, it was not clear to the participant if they are facing pharmacists’ judgment or difficulties due to policy and guidelines. In addition, the business model is another possible contributing factor that must be investigated in the future. Providing counseling and being respectful during patient-pharmacist encounters are referenced as the participants’ primary needs from community pharmacists. Educating patients regarding their health conditions and meeting their needs is already proven critical for positive treatment experiences and subsequent treatment outcomes [47, 48]. Comprehensive substance abuse treatment, which addresses patients’ health and social needs concurrently with pharmacological treatment, leads to a significant reduction in post-treatment substance use and improved patient satisfaction [49–51]. Evidence indicates that applying SUDs preventative measurements rather than palliative care can reduce cost on the health care system [52]. Nevertheless, failing to meet patients’ needs in substance use treatment is a persistent problem [53]. For example, vocational training, child care, transportation, and housing are among the principal needs that have enhanced patients’ treatment outcomes [21], and many treatment programs still lack an adequate referral system.

Also, participants expressed that pharmacists’ attitudes and the stigma toward SUDs were a barrier to proper communication. The stigma toward SUDs, which has been found to exist among health care providers [37], remains a key barrier to accessing health care services and treatment [54–56]. Health care providers, including community pharmacists, should be aware of the stigma, its consequences on people’s physical and mental health, and should try to find new methods to limit its negative impact [57]. In contrast to the many negative encounters, some pharmacists were able to provide a positive experience for participants. Non-judgmental communication was the prevailing characteristic among community pharmacists who provided positive experiences for participants. This finding supports the integral role of social relations and support for the recovery and treatment retention of patients living with SUDs [58, 59].

The following recommendations can be drawn from the current study: there is a need for 1) community pharmacists to become public health advocates and sources of medical advice, 2) training on communication skills for community pharmacists with additional education concerning SUDs and its social elements, and 3) addressing the identified gaps in the policy, dispensing procedures, and practices of the OAT program.

There are several limitations to this study. Firstly, the findings of this study can not be generalized to other settings. However, these findings can give an indication of the needs and barriers people living with SUDs can have in a small urban city like Saskatoon. Also, sampling was voluntary, which may have influenced the outcome of the study. Sampling was mainly through word-of-mouth (snowball sampling); therefore, the sample is not representative of the whole population of people living with SUDs in Saskatoon. Although the study targeted pharmacy clients who use substances, participants were mostly diagnosed with SUDs and enrolled in OAT. Having most of the clients in OAT may have also skewed the data by represented a narrow segment of people who use substances. However, some findings of the study were supported with similar studies in the literature that endorse the support needs and proper exchange of information between people with SUDs and health care providers [17, 20]. In the future, we will investigate the specific needs of subpopulation within SUDs realm, such as injection drug users.

## Conclusion

Community pharmacists have a golden opportunity to deliver preventative and harm reduction interventions. Unfortunately, the experience of patients living with SUDs with community pharmacists is often limited to dispensing medications. Patients’ receptivity for the broader range of services community pharmacists can provide are contingent on respectful communication and genuine, friendly and professional conversations. Health care providers, including community pharmacists, should be trained to address patients’ needs while properly delivering patient-centered care for optimum outcomes. Finally, the role of the business model in shaping pharmacist-SUD patient interactions should be investigated in the future to complement the data gathered in this study.

## Data Availability

The datasets [i.e., focus group transcripts] generated during and analyzed during the current study are not publicly available but are available from the corresponding author on reasonable request.

## Abbreviations

SUDs: Substance Use Disorders
IOM: Institute of Medicine
HIV: Human Immunodeficiency Virus
AIDS: Acquired Immunodeficiency Syndrome
